# Leukocytosis and thrombocytosis after splenectomy: expected finding, infection, or something else: a case report

**DOI:** 10.1186/s13256-024-04744-4

**Published:** 2024-10-17

**Authors:** Nicolas Gonzalez, Jeffry Nahmias, Lisa X. Lee, Matthew Dolich, Michael Lekawa, Allen Kong, Areg Grigorian

**Affiliations:** 1 Division of Trauma, Burns and Surgical Critical Care, Department of Surgery, University of California, Irvine Medical Center, 3800 Chapman Ave, Suite 6200, Orange, Irvine, CA 92868-3298 USA; 2 Division of Hematology/Oncology, Department of Medicine, University of California, Orange, Irvine, CA USA

**Keywords:** Case report, Chronic myeloid leukemia, Trauma, Splenectomy, Leukocytosis, Thrombocytosis

## Abstract

**Background:**

Leukocytosis and thrombocytosis often follow splenectomy in blunt trauma patients, complicating the postoperative identification of infection. While the platelet count to white blood cell ratio provides diagnostic assistance to discern between expected laboratory alterations and infection, diagnoses such as leukemia are often overlooked.

**Case presentation:**

A 53-year-old Hispanic male presented with abdominal pain, nausea, tachycardia, and focal peritonitis 4 days after being assaulted and struck multiple times in the abdomen. Initial white blood cell count was 38.4 × 10^9^/L, platelet count was 691 × 10^9^/L, and lipase was 55 U/L. Computed tomography abdomen/pelvis demonstrated a hematoma encasing the distal pancreas and abutting the stomach and colon. Emergent laparotomy revealed a nearly transected pancreas and devascularized colon, necessitating a distal pancreatectomy, splenectomy, and colonic resection with primary anastomosis. Postoperatively, he had a persistently elevated leukocytosis, thrombocytosis, segmented neutrophils, eosinophilia, and basophilia (peak at 70, 2293, 64, 1.1, and 1.2 × 10^9^/L, respectively). Despite sepsis workup, including repeat computed tomography, no source was identified. Hematology/oncology was consulted for concern for hematologic etiology, with genetic testing and bone marrow biopsy performed. The diagnosis of breakpoint cluster–Abelson gene-positive chronic myeloid leukemia was made based on genetic tests, including polymerase chain reaction and fluorescence in situ hybridization analysis, which confirmed the presence of the Philadelphia chromosome. Bone marrow biopsy suggested a chronic phase. The patient was treated with hydroxyurea and transitioned to imatinib.

**Conclusions:**

Thrombocytosis following splenectomy is a common complication and a plate count to white blood cell count ratio  < 20 indicates infectious etiology. A significantly elevated white blood cell count (> 50 × 10^9^/L) and thrombocytosis (> 2000 × 10^9^/L) may suggest something more ominous, including chronic myeloid leukemia , particularly when elevated granulocyte counts are present. Chronic myeloid leukemia workup includes peripheral smear, bone marrow aspiration, and determination of Philadelphia chromosome. Post-splenectomy vaccines are still indicated within 14 days; however, the timing of immunization with cancer treatment must be considered. Tyrosine kinase inhibitors are the first-line therapy and benefits of pretreatment with hydroxyurea for cytoreduction remain under investigation. Additionally, tyrosine kinase inhibitors have been associated with gastrointestinal perforation and impaired wound healing, necessitating heightened attention in patients with a new bowel anastomosis**.**

## Background

One of the most injured organs in blunt abdominal trauma is the spleen [1]. While nonoperative observation is the standard, splenectomy is necessary in approximately 10–15% of cases [2]. Commonly described postoperative complications include hematoma, pancreatic leak, and infection [3]. Infectious complications occur more commonly in trauma patients undergoing laparotomy with splenectomy compared with without splenectomy [4]. However, post-splenectomy patients commonly exhibit the physiological responses of leukocytosis and thrombocytosis, raising concerns for complications like infection. Specifically, a white blood cell count (WBC) greater than 15 × 10^9^/L at postoperative day (POD) 5 or a platelet count (PC):WBC ratio < 20 are reliable predictors of sepsis following post-traumatic splenectomy [5, 6].

While fever and leukocytosis are primary indicators of infection, these symptoms can be misleading after a splenectomy due to the physiologic response to surgery [4, 6]. This complexity is further compounded in the differential diagnosis by hematologic malignancies such as chronic myeloid leukemia (CML). CML’s pathogenesis is linked to a specific genetic anomaly: the translocation of chromosomes 9 and 22, resulting in the Philadelphia chromosome. This translocation fuses the *Abelson (ABL)* gene on chromosome 9 with the breakpoint cluster region (BCR) gene on chromosome 22, forming the oncogenic *BCR–ABL* fusion gene [7, 8]. Manifestations of CML include marked leukocytosis, an increase in immature granulocytes, basophilia, and eosinophilia in the peripheral blood [7, 8]. Despite being the first cancer associated with a distinct chromosomal abnormality, diagnosing CML remains challenging due to its long chronic asymptomatic phase. This phase can obscure the disease’s presence until significant hematologic abnormalities or clinical symptoms arise, making early detection and diagnosis challenging [7]. In the context of a post-splenectomy patient, the typical physiological leukocytosis and thrombocytosis can mask or mimic the hematologic profiles seen in CML, necessitating a high index of suspicion and thorough diagnostic evaluation to differentiate between a normal postoperative response and a potential malignancy.

In this case report, we discuss a diagnosis of CML made in the context of persistent leukocytosis and thrombocytosis following splenectomy. Given these laboratory findings can indicate leukemia, physicians must maintain a high degree of suspicion to accurately diagnose this relatively rare condition, particularly in the trauma population.

## Case presentation

**Fig. 1:**
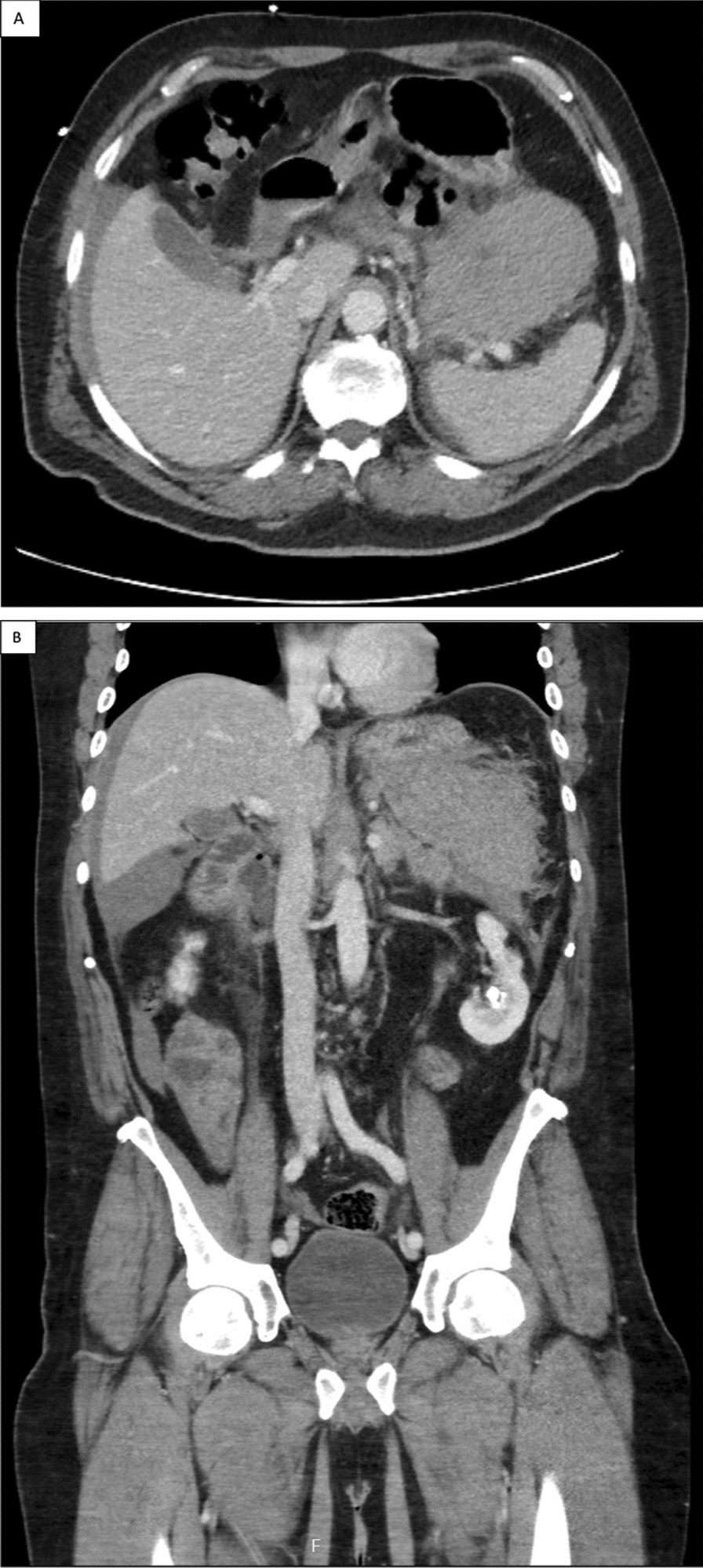
**A** Abdominal computed tomography revealed a 10.3 cm × 6.5 cm × 10.0 cm hematoma partially encasing the pancreatic tail, displacing the stomach anteriorly and medially. **B** Coronal visualization of hematoma

A 53-year-old incarcerated Hispanic male presented from jail with worsening abdominal pain, nausea, vomiting, tachycardia, and focal peritonitis (upper abdominal tenderness with rebound and guarding) 4 days after being struck multiple times in the abdomen. Initial WBC was 38.4 × 10^9^/L, PC was 691 × 10^9^/L, and lipase was 55 U/L. He had a past medical history significant for hypertension and pancreatitis, but no relevant family history. Prior to coming to the hospital, he noticed fatigue, easy bruising, and spontaneous epistaxis with vague abdominal pain for 2 months. A computed tomography (CT) scan of the abdomen demonstrated a hematoma encasing the distal pancreas, abutting the stomach and colon, with a small amount of hemoperitoneum (Fig. 1). Given the concern for bowel injury, the patient was taken for emergent exploratory laparotomy. The surgery revealed a nearly severed distal pancreas and a devascularized colon, necessitating a distal pancreatectomy, splenectomy, and colonic resection with primary anastomosis. The spleen weighed 220 g and measured 11 cm × 8 cm × 5 cm.

Postoperatively, piperacillin/tazobactam was initiated due to delayed presentation with bowel injury. Despite this treatment, he maintained a persistently elevated leukocytosis, thrombocytosis, segmented neutrophils, eosinophilia and basophilia. Leukocytosis peaked at 69.6 × 10^9^/L on POD-9 and thrombocytosis peaked at 2293 × 10^9^/L on POD-12. Antibiotics were continued and aspirin was added for marked thrombocytosis. Despite sepsis workup, including repeat CT with oral and intravenous contrast, no source of infection or contrast leak was identified. His procalcitonin was consistently low (0.11–0.13 ng/mL).

Hematology/oncology was consulted due to concern for hematologic etiology. Peripheral blood smear demonstrated severe leukocytosis and thrombocytosis, but no circulating blasts. Subsequent workup included molecular testing for Janus kinase 2 (JAK2), calreticulin (CALR), and myeloproliferative leukemia virus oncogene (MPL), which were negative. Polymerase chain reaction (PCR) of *BCR–ABL* was positive. A subsequent bone marrow biopsy demonstrated slightly hypercellular marrow (70%) with evidence of trilineage hematopoiesis, mild myeloid hyperplasia, mild megakaryocytic hyperplasia, and molecular evidence of *BCR/ABL* + fusion, consistent with CML, chronic phase. The *BCR–ABL1* quantitative analysis reported an international scale (IS) value of 24.6235%. The *BCR–ABL* positivity was confirmed through reverse transcription quantitative polymerase chain reaction (RT–qPCR), which detected the e14a2 (p210) transcript. The fluorescence in situ hybridization (FISH) analysis detected *BCR–ABL1* fusions in 91.5% of the nuclei examined, confirming the presence of the t(9;22) translocation (Philadelphia chromosome). Flow cytometry showed no increased blasts (< 1%). By immunohistochemistry, CD34-positive blasts were estimated at 1% of all cells. The patient’s Sokal score was 0.8, placing him in the intermediate category. Similarly, the EUTOS long-term survival (ELTS) score was 1.6, also indicating an intermediate category.

Initial treatment included hydroxyurea, with transition to imatinib (400 mg daily). Infectious disease specialists recommended post-splenectomy vaccinations, including Prevnar 20, HiB, MenB (2 doses at least 1 month apart), and Menveo 2 doses (each at least 2 months apart), alongside prophylactic azithromycin until completion of the vaccine series. After initiation of CML treatment, the patient’s hospital course was further complicated by intra-abdominal fluid collections, necessitating medical therapy via cefepime, metronidazole, and micafungin, as well as direct drainage by interventional radiology. He was then discharged to an outside facility with WBC of 11.6 × 10^9^/L and PC of 932 × 10^9^/L.

At outpatient follow-up, he demonstrated appropriate recovery by tolerating a diet with regular bowel movements while continuing imatinib. A period of 1 year later, he continues daily imatinib therapy. He reports he is doing quite well and has not experienced any adverse effects.

## Discussion

The PC and WBC count can be further analyzed to provide a differential diagnosis for post-splenectomy hematologic derangements. Reactive thrombocytosis, seen in 75% of patients, results from the spleen’s role in platelet sequestration and clearance [5]. Similarly, transient leukocytosis is commonly exaggerated. The PC:WBC provides diagnostic assistance to discern between expected laboratory alterations and infection. Specifically, PC:WBC < 20 indicates infectious etiology based upon the premise that thrombocytosis is an expected physiologic response to splenectomy, while thrombocytopenia is often a response to widespread infection through increased platelet adhesiveness and consumption [4, 5, 9]. Previous retrospective studies have identified a combination of an injury severity score > 16, WBC 15 × 10^9^/L and PC:WBC < 20 as having 96% predictive accuracy for sepsis in post splenectomy trauma patients [3, 9]. However, marked leukocytosis (> 50 × 10^9^/L) and thrombocytosis (> 2000 × 10^9^/L), especially when accompanied by increased granulocyte counts, may suggest something more [6].

Early detection remains a challenge in CML management, with 50% of cases diagnosed incidentally through routine laboratory testing [7]. Although CML symptoms can be vague, splenomegaly, occurring in up to 75% of cases, may increase susceptibility to splenic injury in blunt abdominal trauma [7, 10]. Standard workup includes peripheral smear, determination of *BCR–ABL* via FISH or PCR, and bone marrow aspiration. The peripheral smear may help distinguish oncologic etiology of leukocytosis from a leukemoid reaction. In CML, the WBC differential shows all cells of the granulocyte lineage including eosinophils, basophils, possibly blasts and early precursor neutrophils. In leukemoid reactions, mainly mature neutrophils are seen with slight “left shift” in differentiation. In addition, within the neutrophils, one may see evidence of toxic granulations or Dohle bodies. Finally, leukocyte alkaline phosphatase (LAP) is a cytochemical stain that differentiates abnormal neutrophils as seen in CML versus normal neutrophils in reactive processes leading to another differentiation tool called the LAP score. Bone marrow analysis is crucial for staging, as the prevalence of blasts or basophils distinguishes between chronic (CP), accelerated, and blastic phases [11]. Identifying progression from CP, the most common and favorable prognosis stage, to more advanced stages is vital for prognosis and treatment adjustments, although clinical boundaries remain ill-defined [11].

The identification of the *BCR–ABL* oncogene allowed for the evolution of targeted therapy through tyrosine kinase inhibitors (TKIs), which ultimately block cellular proliferation [12]. TKIs revolutionized the natural history of CML by increasing overall survival, resulting in life expectancy nearly equal to that of the general population [7, 12]. Hydroxyurea functions to reduce WBC counts and can be utilized in the acute setting to reduce very elevated leukocytosis or thrombocytosis for most leukemias, including CML when CML is suspected but unconfirmed [10]. In practice, hydroxyurea is discontinued once the presence of the Philadelphia chromosome is confirmed and a TKI is initiated [10]. Imatinib, a first-generation TKI, shows a 65–70% probability of complete cytogenic remission at 12 months, with significant reduction in disease burden over longer periods [13]. Second-generation TKIs such as nilotinib and dasatinib offer quicker and deeper responses than imatinib, though their long-term survival impact remains under evaluation. Current prescribing trends in the USA favor imatinib, used in nearly 60% of cases [12, 14].

Thrombocytosis in isolation following splenectomy poses a unique challenge. The link between splenectomy and venous thromboembolism remains debatable, but thrombocytosis, especially when PC exceeds 600 × 10^9^/L, may increase the risk of thrombotic complications [15]. First line therapy therefore involves antiplatelet medications. In instances of extreme thrombocytosis accompanied by evidence of arterial or venous thrombosis, hydroxyurea or anagrelide have been suggested for their cytoreductive effects [16, 17]. Therefore, if thrombocytosis is present in the setting of CML, bridging hydroxyurea prior to TKI initiation may be sufficiently therapeutic, barring the need to initiate a specific antiplatelet agent.

While the direct targeting of TKIs proves advantageous, these drugs are not without risks. TKIs have been associated with off target effects including hemorrhage, gastrointestinal perforation, fistula formation, and impaired tissue healing [18, 19]. Therefore, surgeons must be vigilant for signs of perforation or leakage in patients who have recently undergone bowel anastomosis and are on TKI therapy. These complications have been observed in clinical trials, but detailed data on their incidence is scarce due to the lack of comprehensive studies. Research in mice has shown that systemic treatment of imatinib mesylate can delay wound closure and the formation of granulation tissue by inhibiting platelet-derived growth factor [19]. Drug labels recommend TKI discontinuation prior to elective surgery and the only absolute contraindication being gastric perforation [18, 19]. Since there is limited clinical data regarding the timing of reinitiation of TKIs following surgery, the decision is based on clinical judgement.

The timing of post-splenectomy vaccinations is a critical consideration in patients with a new diagnosis of CML, especially given the spleen’s role in immunity against encapsulated bacteria. Vaccination against *Streptococcus pneumoniae*, *Haemophilus influenza* type B, and *Neisseria meningitides* are crucial to prevent overwhelming post-splenectomy infection, a condition with a high mortality rate [20]. In emergent splenectomy cases, vaccines should be administered after trauma [21]. In patients undergoing chemotherapy, vaccines must be administered at least 2 weeks before treatment or 3 months after [22]. However, for CML patients undergoing TKI therapy, the response to vaccines can be significantly impaired. Studies have suggested that TKIs might reduce the efficacy of vaccines by affecting memory B cell subsets and possibly T cell responses [23, 24]. Therefore, in the context of a new CML diagnosis and subsequent TKI therapy after trauma splenectomy, careful planning of the vaccination schedule is essential, considering the potential interactions between the disease treatment and the immune response to vaccines. Additionally, a period of bridging with antibiotics against encapsulated organisms may be necessary. Ultimately, involving an infectious disease specialist may be prudent.

## Conclusion

This case report describes an adult male who presented after blunt abdominal trauma requiring a splenectomy, exhibiting significant and persistent leukocytosis and thrombocytosis. These findings were later attributed to previously undiagnosed and untreated CML. This case is noteworthy because, while splenectomy typically leads to distinct hematologic changes, the possibility of leukemia as an underlying cause is seldom considered, highlighting the need for a thorough differential diagnosis in similar clinical scenarios.

## Data Availability

Not applicable.

## Abbreviations

WBC: White blood cell
POD: Post-operative day
PC: Platelet count
CML: Chronic myeloid leukemia
BCR: Breakpoint cluster region
ABL: Abelson
CT: Computed tomography
JAK2: Janus Kinase 2
CALR: Calreticulin
MPL: Myeloproliferative leukemia virus oncogene
IS: International scale
RT–qPCR: Reverse transcription quantitative polymerase chain reaction
FISH: Fluorescence in situ hybridization
PCR: Polymerase chain reaction
LAP: Leukocyte alkaline phosphatase
CP: Chronic phase
TKI: Tyrosine kinase inhibitor
